# Association between obesity and magnetic resonance imaging defined patellar tendinopathy in community-based adults: a cross-sectional study

**DOI:** 10.1186/1471-2474-15-266

**Published:** 2014-08-07

**Authors:** Jessica Fairley, Jason Toppi, Flavia M Cicuttini, Anita E Wluka, Graham G Giles, Jill Cook, Richard O’Sullivan, Yuanyuan Wang

**Affiliations:** 1 School of Public Health and Preventive Medicine, Monash University, Alfred Hospital, Melbourne VIC 3004, Australia; 2 Centre for Epidemiology and Biostatistics, Melbourne School of Population and Global Health, The University of Melbourne, Carlton VIC 3053, Australia; 3 Cancer Epidemiology Centre, Cancer Council Victoria, Carlton VIC 3053, Australia; 4 Department of Physiotherapy, School of Primary Health Care, Faculty of Medicine, Nursing and Health Sciences, Monash University, Frankston VIC 3199, Australia; 5 Healthcare Imaging Services, MRI Department, Epworth Hospital, Richmond VIC 3131, Australia; 6 Department of Medicine, Central Clinical School, Monash University, Melbourne, Australia

**Keywords:** Obesity, Patellar tendinopathy, Body mass index, Weight, Fat mass, Magnetic resonance imaging

## Abstract

**Background:**

Patellar tendinopathy is a common cause of activity-related anterior knee pain. Evidence is conflicting as to whether obesity is a risk factor for this condition. The aim of this study was to determine the relationship between obesity and prevalence of magnetic resonance imaging (MRI) defined patellar tendinopathy in community-based adults.

**Methods:**

297 participants aged 50–79 years with no history of knee pain or injury were recruited from an existing community-based cohort. Measures of obesity included measured weight and body mass index (BMI), self-reported weight at age of 18–21 years and heaviest lifetime weight. Fat-free mass and fat mass were measured using bioelectrical impedance. Participants underwent MRI of the dominant knee. Patellar tendinopathy was defined on both T1- and T2-weighted images.

**Results:**

The prevalence of MRI defined patellar tendinopathy was 28.3%. Current weight (OR per kg = 1.04, 95% CI 1.01-1.06, P = 0.002), BMI (OR per kg/m^2^ = 1.10, 95% CI 1.04-1.17, P = 0.002), heaviest lifetime weight (OR per kg = 1.03, 95% CI 1.01-1.05, P = 0.007) and weight at age of 18–21 years (OR per kg = 1.03, 95% CI 1.00-1.07, P = 0.05) were all positively associated with the prevalence of patellar tendinopathy. Neither fat mass nor fat-free mass was associated with patellar tendinopathy.

**Conclusion:**

MRI defined patellar tendinopathy is common in community-based adults and is associated with current and past history of obesity assessed by BMI or body weight, but not fat mass. The findings suggest a mechanical pathogenesis of patellar tendinopathy and patellar tendinopathy may be one mechanism for obesity related anterior knee pain.

## Background

Patellar tendinopathy is a clinical condition causing activity-related anterior knee pain and subsequent dysfunction [1,2]. It has been most commonly described in athletes participating in sports that involve jumping, earning it the name “jumper’s knee”, where the tendon matrix is affected by repetitive stress on the tendon [3,4], but patellar tendinopathy has also been found in people who do not participate in jumping sports [3,5]. The prime pathological feature of patellar tendinopathy histologically is characterised by mucoid degeneration which can be detected using ultrasonography or magnetic resonance imaging (MRI) [6-8]. Although not all individuals with evidence of patellar tendinopathy on imaging have symptoms [6,9], those tendon abnormalities identified by imaging modality in asymptomatic individuals predict the development of tendon-related symptoms and disability [10,11]. In addition, these imaging changes are not always present in those with symptomatic patellar tendinopathy. This may in part be due to the incorrect attribution of symptoms to patellar tendinopathy when other joint pathology may be the source of symptoms [12,13]. MRI has advanced our understanding of factors that contribute to pain and function in the tibiofemoral joint [14,15]. These structural changes in and around the knee joint develop on a continuum from the healthy joint to the diseased joint.

Pathology in the patellofemoral compartment is increasingly recognised as a source of pain and impaired function with aging [16,17]. However, there has been relatively little work at the patellofemoral joint, compared with the tibiofemoral joint, examining structures such as tendons. Most of the instruments examining pain and function in epidemiological and clinical studies of older people focus on the tibiofemoral compartment and do not address symptoms that may be due to patellar tendinopathy, such as activity-related anterior knee pain through important functional activities such as knee bending.

A large body of work on patellar tendinopathy performed using athletic populations has identified male gender [18-20] and body mass [3,21,22] as risk factors. The evidence is conflicting about any association between patellar tendinopathy and obesity, a condition which is known to increase strain on joints and tendons throughout the body, especially the weight-bearing joints, with some studies showing no association [18,23]. Any association between body composition and risk of patellar tendinopathy is also inconclusive [21,23]. In older populations, obesity has been related to knee pain and poor function [24,25]. It is possible that patellar tendon pathology may be one of the mechanisms for obesity related anterior knee pain in older people.

Thus the aim of the current study was to determine the prevalence of MRI defined patellar tendinopathy in community-based asymptomatic adults and whether it is associated with obesity and body composition. Examining this in an asymptomatic population may reduce the potential for any associations identified being due to reverse causation. We hypothesised that obesity would be associated with increased risk of MRI defined patellar tendinopathy in community-based individuals.

## Methods

### Study participants

The study was conducted within the Melbourne Collaborative Cohort Study (MCCS), a prospective cohort study of 41,514 residents of Melbourne, Australia. The aim of the MCCS is to examine the role of lifestyle and genetic factors in the risk of cancer and chronic diseases [26]. Participants for the current study were recruited from within the MCCS between 2003–2004 as described [27]. Briefly, participants were eligible if they were aged between 50–79 years without any of the following exclusion criteria: a clinical diagnosis of knee osteoarthritis as defined by American College of Rheumatology criteria [28]; knee pain lasting for > 24Â hours in the last 5Â years; a previous knee injury requiring non-weight bearing treatment for > 24Â hours or surgery (including arthroscopy); or a history of any form of arthritis diagnosed by a medical practitioner. A further exclusion criterion was a contraindication to MRI including pacemaker, metal sutures, presence of shrapnel or iron filings in the eye, or claustrophobia. The study was approved by The Cancer Council Victoria’s Human Research Ethics Committee and Monash University Human Research Ethics Committee. All participants gave written informed consent.

### Anthropometric measurements

At baseline (2003–2004), height (cm) was measured using a stadiometer with shoes removed, and weight (kg) was measured with bulky clothing removed. Body mass index (BMI) was calculated from these data [weight (kg)/height^2^ (m^2^)]. Bioelectrical impedance analysis was performed with a single frequency (50Â kHz) electric current produced by a BIA-101A RJL system analyser (RJL systems, Detroit, MI). Resistance and reactance were measured with participants in a supine position. Non-adipose mass, hereafter termed fat-free mass, was estimated as 9.1536 + (0.4273 Ã— height^2^/resistance) + (0.1926 Ã— weight) + (0.0667 Ã— reactance) for males, and 7.7435 + (0.4542 Ã— height^2^/resistance) + (0.119 Ã— weight) + (0.0455 Ã— reactance) for females [29]. Adipose mass, hereafter termed fat mass, was subsequently calculated as weight – fat-free mass. There is evidence for the validity and reliability of bioelectrical impedance analysis in determining body composition [30,31]. A particular issue for bioelectric impedance analysis is the absence of a standard equation to estimate fat-free mass. We chose a formula developed using subjects of similar ethnicity, age, and BMI distribution to the MCCS population [29] and validated using sound statistical techniques. At MCCS baseline (1990–1994), participants were also asked to recall their weight when they were aged between 18–21 years and their heaviest lifetime weight.

### MRI acquisition

An MRI of the dominant knee (defined as the lower-limb from which the participant stepped off from when initiating gait) was performed between October 2003 and December 2004 and repeated approximately 2Â years later. Knees were imaged in the sagittal plane on a 1.5-T whole body magnetic resonance unit (Gyroscan Intera, Philips Medical Systems, Eindhoven, The Netherlands) using a commercial transmit-receive extremity coil. The following sequence and parameters were used: a T_1_-weighted fat suppressed 3D gradient recall acquisition in the steady state; flip angle 55 degrees; repetition time 58Â msec; echo time 12Â msec; field of view 16Â cm; 60 partitions; 512 Ã— 512 matrix; one acquisition time 11Â min 56Â sec. Sagittal images were obtained at a partition thickness of 1.5Â mm and an in-plane resolution of 0.31 Ã— 0.31Â mm (512 Ã— 512Â pixels). In addition, a coronal T_2_-weighted fat-saturated acquisition, repetition time 3500–3800Â msec, echo time 50Â msec, with a slice thickness of 3.0Â mm, a 1.0Â mm interslice gap, 1 excitation, a field of view of 13Â cm, and a matrix of 256 Ã— 192Â pixels was also obtained.

### Assessment of patellar tendinopathy

Patellar tendinopathy was defined as an area of increased signal intensity of characteristic pattern, size and distribution on at least two adjacent slices in the proximal region of the inferior patellar tendon. Two trained observers, who were blinded to participant characteristics, assessed the presence of lesions for each participant in T1-weighted fat-saturated sagittal images. T2-weighted fat-saturated coronal images were used to confirm the presence of patellar tendinopathy and ensure a magic angle effect was not contributing to positive results. When echo time is short, increased signal intensity can be observed in the absence of pathology, which is known as the magic angle effect. To overcome the magic angle effect a T2-weighted sequence can be applied [32], which was done in this study. The patellar tendon was graded as either â€˜definite tendinopathy’ or â€˜no tendinopathy’ [9,32,33] (FigureÂ 1). The reproducibility for determination of definite patellar tendinopathy was assessed using 50 randomly selected knee MRIs (intraclass coefficient correlation was 0.94).

**Figure 1:**
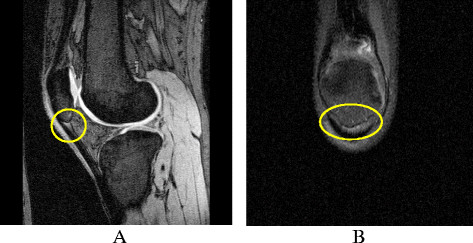
**Patellar tendinopathy on MRI on A.** T1-weighted fat-saturated sagittal MRI; and **B**. T2-weighted fat-saturated coronal MRI.

### Statistical analysis

The outcome measure was the prevalence of MRI defined patellar tendinopathy. The exposures of interest were different body mass measurements (weight, BMI, and body composition). Binary logistic regression was used to examine the association between body mass measures and the prevalence of MRI defined patellar tendinopathy, adjusting for confounders of age, gender, and height (for body weight only). A p-value of less than 0.05 (two-tailed) was regarded as statistically significant. All analyses were performed using IBM SPSS version 20.

## Results

The characteristics of the 297 participants aged between 50 and 79Â years (mean 58.0Â years, SD 5.5Â years) are presented in TableÂ 1. Participants with patellar tendinopathy (n = 84, 28.3%) were more likely to be men (P < 0.001), taller (P = 0.02), and had greater body weight (P < 0.001) and BMI (P = 0.001) than those without patellar tendinopathy. Weight at the age of 18–21 years and heaviest lifetime weight were also greater for those with tendinopathy compared with those without (all P < 0.001). Two hundred and thirty three (78.5%) participants had current fat mass and fat free mass measures. Participants with patellar tendinopathy had higher fat free mass than those without (P < 0.001), but there was no significant difference in fat mass between the two groups (P = 0.21).

**Table 1:** Characteristics of study population

	**No tendinopathy (n = 213)**	**Tendinopathy (n = 84)**	P value*
Age (years)	57.6 (5.2)	59.0 (6.0)	0.06
Female, number (%)	147 (69)	39 (46)	<0.001
Height (cm)	167.3 (8.7)	170.0 (9.5)	0.02
Weight (kg)	71.3 (13.8)	78.9 (14.2)	<0.001
Body mass index (kg/m^2^)	25.4 (4.2)	27.2 (4.2)	0.001
Fat-free mass (kg)^â€ ^	44.4 (9.3)	51.7 (10.7)	<0.001
Fat mass (kg)^â€ ^	26.1 (9.0)	27.8 (8.4)	0.21
Weight at ages of 18–21 years (kg)	59.2 (11.1)	64.3 (11.3)	<0.001
Heaviest lifetime weight (kg)	71.1 (14.1)	78.7 (13.7)	<0.001

Values are reported as mean (SD) except for percentages.

*Differences between those with and without patellar tendinopathy, using independent samples t test or Chi-squared test.

^**â€ **^Data available on 233 participants.

### Relationship between weight and BMI and prevalence of patellar tendinopathy

In univariate analyses, BMI, body weight, weight at ages of 18–21 years and heaviest lifetime weight were all positively associated with the prevalence of patellar tendinopathy (TableÂ 2). After adjusting for age and gender, BMI remained positively associated with the prevalence of patellar tendinopathy (odds ratio (OR) 1.10, 95% confidence interval (CI) 1.04 - 1.17). Body weight (OR 1.04, 95% CI 1.01 - 1.06) and heaviest lifetime weight (OR 1.03, 95% CI 1.01 - 1.05) also remained positively associated with the prevalence of patellar tendinopathy after adjusting for age, gender and height. Weight at ages of 18–21 years was marginally associated with the prevalence of patellar tendinopathy (OR 1.03, 95% CI 1.00 - 1.07) (TableÂ 2).

**Table 2:** Associations of weight and body mass index with prevalence of patellar tendinopathy

	**Univariate analysis**		**Multivariate analysis**	
	**Odds ratio (95% CI)**	**P value**	**Odds ratio (95% CI)**	**P value**
Body mass index (kg/m^2^)^1^	1.10 (1.04, 1.17)	0.001	1.10 (1.04, 1.17)	0.002
Weight (kg)^2^	1.04 (1.02, 1.06)	<0.001	1.04 (1.01, 1.06)	0.002
Weight at ages of 18–21 years (kg)^2^	1.04 (1.02, 1.07)	0.001	1.03 (1.00, 1.07)	0.05
Heaviest lifetime weight (kg)^2^	1.04 (1.02, 1.06)	<0.001	1.03 (1.01, 1.05)	0.007

^1^adjusting for age and gender.

^2^adjusting for age, gender, and height.

### Relationship between body composition and prevalence of patellar tendinopathy

In univariate analyses, fat free mass was positively associated with the prevalence of patellar tendinopathy but there was no significant association with fat mass (TableÂ 3). After adjusting for age and gender, the significant association with fat free mass remained (OR 1.08, 95% CI 1.01 - 1.15) while fat mass was weakly associated with the prevalence of patellar tendinopathy (OR 1.03, 95% CI 1.00 - 1.07). After adjustment for fat free mass and fat mass along with age and gender, neither fat free mass (OR 1.07, 95% CI 0.99 - 1.16) nor fat mass (OR 1.01, 95% CI 0.96 - 1.05) were significantly associated with the prevalence of patellar tendinopathy (TableÂ 3).

**Table 3:** Associations between body composition and prevalence of patellar tendinopathy

	**Univariate analysis**		**Multivariate analysis**^**1**^		**Multivariate analysis**^**2**^	
	**Odds ratio (95% CI)**	**P value**	**Odds ratio (95% CI)**	**P value**	**Odds ratio (95% CI)**	**P value**
Fat free mass (kg)	1.07 (1.04, 1.10)	<0.001	1.08 (1.01, 1.15)	0.02	1.07 (0.99, 1.16)	0.10
Fat mass (kg)	1.02 (0.99, 1.05)	0.21	1.03 (1.00, 1.07)	0.08	1.01 (0.96, 1.05)	0.78

^1^adjusting for age and gender.

^2^adjusting for age, gender, fat free mass and fat mass.

## Discussion

In community-based adults without clinical knee disease, MRI defined patellar tendinopathy was common (28.3%). There was an increased risk of MRI defined patellar tendinopathy associated with both current and past history of obesity as assessed by either BMI or body weight. Neither fat mass nor fat free mass was associated with the risk of MRI defined patellar tendinopathy when they were co-adjusted.

Existing studies aimed at determining the prevalence of patellar tendinopathy have used young athletic populations, both elite and non-elite. Patellar tendinopathy, which is also known as “jumper’s knee”, is traditionally considered to be mainly a disease of athletes, especially those participating in sports involving significant force through the patellar tendon [2]. The prevalence and significance of patellar tendinopathy have been illustrated in a number of samples of athletes from different sports, with prevalence ranging from 2.5% to 41% [3,20,34]. Data are limited regarding the prevalence of patellar tendinopathy in non-athletic, healthy middle-aged to older populations. Our study demonstrated that the prevalence of MRI defined patellar tendinopathy was 28.3% in a community-based population of adults without clinical knee disease, supporting the findings from a previous study that prevalence of patellar tendinopathy is increased in older populations [35].

There is conflicting evidence for an association between obesity and patellar tendinopathy. A clinical diagnosis of patellar tendinopathy has been reported to be associated with increased weight [3,21,22] and BMI [21,22] by a number of studies of athletes, but there is also evidence suggesting that weight is not a significant risk factor for patellar tendinopathy [18,23]. Our study has demonstrated a clear relationship between current and past history of obesity and MRI defined patellar tendinopathy in an asymptomatic older population. The value of examining a population without clinical symptoms is important since it is unlikely that the knee pain and function limitation cause reduced mobility and increased obesity, i.e. the potential reverse causality. It is more likely that obesity contributes to these pathological tendon abnormalities. Thus we highlight the unique contribution of our results from an unselected middle-aged population in contributing to knowledge of patellar tendinopathy.

We found no relationship between body composition (fat mass and fat free mass) and MRI defined patellar tendinopathy when adjusting for age, gender, fat mass and fat free mass. This is supported by the findings from a previous study that there is no clear link between body composition and patellar tendinopathy in elite female basketball players [23]. On the other hand, another study of competitive volleyball players reported an association between central obesity assessed by waist circumference and the risk of patellar tendinopathy [21]. The reasons for the discrepancy of findings are not known, but may be due to different study populations (different age group, gender, and athletes vs. non-athletes) and different methods used to assess body composition and patellar tendinopathy. Further investigations are needed.

Our findings of a positive association between obesity (past and present) and MRI defined patellar tendinopathy and no association with body composition suggest a mechanical effect of obesity on patellar tendinopathy. This mechanism is supported by existing literature that patellar tendinopathy is a disease of athletes who have repeated heavy loading through the patellar tendon, especially sports involving jumping. Differences in prevalence have been established for different sporting populations [3,20,34]; the interpretation being that the degree of tendon strain varies between different sports. Previous studies have shown a direct relationship between the number of training sessions and physically demanding work and the risk of patellar tendinopathy [23,36,37]. It is thus of interest that increased mechanical load through the knee due to obesity as assessed by either BMI or body weight is significantly associated with the risk of patellar tendinopathy. This is consistent with the histological basis for patellar tendinopathy which shows matrix changes without any inflammatory component [6,35].

The patellofemoral compartment is being increasingly recognised as a major source of pain and disease [16,17]. Patellar tendinopathy can be debilitating and cause significant morbidity [38]. Symptomatic patellar tendinopathy may have a significant negative impact on activity, quality of life and ultimately mental health [4,39]. Although a standardised patellar tendon outcome measure has been developed and extensively validated (Victorian Institute of Sport Assessment, VISA) [40], most traditional and validated instruments for assessing knee pain (such as the Western Ontario and McMaster Universities Osteoarthritis Index (WOMAC) scoring system [41]) focus on the tibiofemoral compartment rather than the patellofemoral compartment. It is well recognized that functional limitations in activities such as knee bending, which have significant impact on knee function, are common in older people. Pathology in the patellofemoral compartment has a significant impact on pain and quality of life [16,17]. Whether symptoms in the region are due to joint pathology or other structures such as patellar tendinopathy has not been examined. Our study suggests that MRI defined patellar tendinopathy is common in older people. Using a non-specific tool (WOMAC), we found some trends for worse knee functions in older people with MRI defined patellar tendinopathy compared with those without (data not shown). However, further work using specific tools will be needed to determine whether patellar tendinopathy explains some of the anterior knee pain and common functional abnormalities seen in older people but not explored by commonly used knee instruments.

This study has limitations. Although the cross-sectional design enabled us to generate hypotheses and add to current evidence using a reasonably sized population, the findings of this cross-sectional study need to be confirmed by longitudinal studies. Further investigation will be required to detail the natural history and other contributing factors for patellar tendinopathy, particularly clarifying whether the disease process has been there since early adulthood or whether it has developed later in life. The participants were asked to recall their weight at 18–21 years at the MCCS inception (1990–1994) when they were aged 40–69 years. Self-report of past weight over a similar period to our study has shown moderate to strong correlation with measured weight (correlation coefficients range from 0.64 to 0.95), and these correlations are modified by sex and current weight [42-44]. Moreover, the reliability of self-reported past weight is generally supported in epidemiologic studies [42-44]. The main strength of the study is that patellar tendinopathy has been measured using a non-invasive MRI-based method from both T1- and T2-weighted images to encounter the magic angle effect which showed high reproducibility. In this study we recruited participants with no history of knee disease from a community-based cohort. However we did not specifically ask about knee pain on bending, which would be more specific for patellar tendinopathy. Given the participants did not have significant knee pain, it is unlikely that knee pain restricted activity sufficiently to contribute to the obesity.

## Conclusions

Our study highlights a significant prevalence of MRI defined patellar tendinopathy of 28.3% in a sample of community-based adults. The positive association of MRI defined patellar tendinopathy with both current and past obesity, but not body composition, suggests a mechanical mechanism. The contribution of MRI defined patellar tendinopathy on knee symptoms associated with obesity in the older people warrants further investigation.

## Competing interests

The authors declare that there are no competing interests.

## Authors’ contributions

JF was involved in data collection, performed data analysis and interpretation, and drafted the manuscript. JT was involved in data collection and interpretation. FMC was involved in conception and design of the study and data interpretation. AEW was involved in data interpretation. GGG was involved in data collection. JC was involved in data interpretation. RO oversaw imaging and was involved in data collection. YW was involved in conception and design of the study, data analysis and interpretation, and coordinated all suggestions and edits. All authors participated in reviewing and editing the manuscript, and approved the final manuscript.

## Pre-publication history

The pre-publication history for this paper can be accessed here:

http://www.biomedcentral.com/1471-2474/15/266/prepub
